# The Mechanism of Action of Antimicrobial Peptides: Lipid Vesicles vs. Bacteria

**DOI:** 10.3389/fimmu.2012.00236

**Published:** 2012-08-02

**Authors:** Manuel N. Melo, Miguel A. R. B. Castanho

**Affiliations:** ^1^Groningen Biotechnological and Biomolecular Institute, University of GroningenGroningen, Netherlands; ^2^Institute of Molecular Medicine, School of Medicine, University of LisbonLisbon, Portugal

## Overview

### AMPs and their MICs

Antimicrobial peptides (AMPs) are a class of antibiotics that is part of the innate immune system of virtually all organisms. This is a broadly defined class, with many common characteristics – and many exceptions to those. The reader will find detailed reviews of such characteristics and proposed subclassifications of the AMPs (see Yeaman and Yount, 2003, for example) but, very generally, these are short, cationic peptides that kill bacteria (sometimes only Gram-positives, sometimes only Gram-negatives, sometimes both) at concentrations typically in the low micromolar range. The clinical interest of some AMPs stems from a low toxicity to mammalian cells, together with the fact that bacterial resistance to these antibiotics seems inherently difficult to acquire (Bell and Gouyon, 2003; Perron et al., 2006).

Of importance to the discussion later on is the method and conditions through which MICs are established for a given peptide against a given bacterial strain. A widely adopted protocol to this end is the one provided by the lab of Robert Hancock (Giacometti et al., 2000); it consists in monitoring the growth of quite dilute bacterial suspensions after administration of different peptide doses. The MIC*n* is then defined as the peptide concentration that causes a reduction of *n*% in growth relative to control (MIC_50_ and MIC_90_ being the most commonly reported) after a set time interval.

### Liposomes as biophysical membrane models

AMPs make good test subjects for biophysical methodologies. Accounting for this is the peptides’ small size, and the ease with which one can influence their molecular-level properties simply by changing the amino acid sequence. In addition, an early recognition of the importance of membrane interaction and disruption for the action of AMPs (Lehrer et al., 1989) allowed the coupling of the mature field of membrane biophysics to study the latter.

With the exception of certain methodologies, lipid vesicles have been the preferred biophysical model for mimicking biological membranes – bacterial or otherwise (Matsuzaki et al., 1995; Willumeit et al., 2005). In the three decades of AMP studies a wealth of biophysical data has been collected from these peptide-vesicle systems. These data include structural features – such as peptide structuring, oligomerization, depth of insertion, etc. – thermodynamic properties – such as peptide-membrane affinity and functional aspects – typically membrane disruption events such as poration or lysis, but also peptide translocation and lipid charge neutralization (Shai, 1999).

### Membrane disruption and high peptide-to-lipid ratios

It came to our attention, first from our own studies and then from others’ reports (Melo et al., 2009), that several membrane disruptive events were often observed at concentrations where the vesicle membrane would be almost completely covered by the peptide–peptide-to-lipid (P:L) ratios higher than 1:25. It is common to read remarks on the unphysiological character of results obtained using too high such ratios (Zhang et al., 2001; Hancock and Rozek, 2002; Nicolas, 2009) but a sound basis for this assumption is yet to be provided.

It is easy to see the reasons behind the intuitive notion of the unphysiological character of high P:L ratios: first, peptides are nanometer-scale entities, typically present at micromolar concentrations, that seem impossible to exist in enough numbers to cover a (relatively) macroscopic entity such as a bacterium. Second, laboratory research often requires the use of peptide and lipid concentrations equivalent to many times those present in MIC assays; this further contributes to the notion that any events thus observed are only achievable with the extreme concentrations available *in vitro*, even though the bound P:L ratios may actually be the same as in much more dilute conditions.

Conversely to these considerations, already in 2000 Tossi et al. had pointed out the great excess of peptide to bacterial lipids at typical AMP MIC conditions. We further developed that consideration by taking into account measured affinities of AMPs to bacterial membrane mimics. From there we arrived at expected peptide-to-lipid ratios in the bacterial membrane that fall precisely in the range where liposomal disruption is commonly observed (Melo et al., 2009, 2011), and that many insist on calling “unphysiological.” Furthermore, attempts at directly quantifying bacterium-bound AMPs – though scarce – again point to very high degrees of bacterial surface coverage (Steiner et al., 1988; Albrecht et al., 2002; Tran et al., 2002; Ding et al., 2003).

## From Biophysics to Biology

### Bacterial membrane – how much of it is there?

One of the main issues when trying to assess the validity of high P:L ratios comes from the fact that it is not readily measurable how much membrane a bacterial suspension has available for interaction. To estimate a peptide-to-lipid proportion Tossi et al. needed to find an approximate value for this amount of available lipid. They did so using a geometrical approach, taking into account the average areas of the bacterial surface and of a membrane phospholipid. After factoring in the bacterial titer a lipid concentration of 25 nM was estimated (Tossi et al., 2000). Blazyk et al. (2001) later followed a similar reasoning, estimating a value of 66 nM. We carried out a different calculation, using the bacterial dry weight, its fraction that are phospholipids, and an average phospholipid mass. The value of 58 nM to which we arrived (Melo et al., 2011) is in good agreement with the two previous estimates.

The bottom line from these numbers is that under MIC assay conditions membrane lipids are present in concentrations in the range of tens of nanomolars, whereas there are about two orders of magnitude more peptide available to bind it.

### How much peptide binds?

Not all the peptide in solution will bind the available membrane lipids, as binding is a reversible process subject to an equilibrium constant (Santos et al., 2003). How much peptide does bind can be quantitatively calculated since those equilibrium constants are often measured, usually in the form of membrane binding or partition (*K_p_*) constants. We have shown (Melo et al., 2011) that typical partition constants for AMPs, though quite high, will drive less than 1% of the total peptide to the membrane; this is still enough, however, for a typical AMP to reach P:L ratios between 1:20 and 1:10 at global concentrations close to their MIC. These calculations provide theoretical significance to the aforementioned observations of disruptive events at high P:L ratios. This is not to say that all AMPs only become active at high degrees of membrane coverage, but it is plausible to say that many might behave thus, and therefore that no observations at high P:L ratios should be discarded.

### Bridging the GAP: MIC prediction

The simple relationships that were used to establish the plausibility of high P:L ratios could be used in the reverse direction: by knowing a critical P:L ratio at which an AMP becomes disruptive against a given membrane model we can calculate the global AMP concentration required for it to reach a similar P:L ratio in a bacterium. This concentration will be a MIC estimate. A very simple equation (Melo et al., 2011) summarizes the relationship between MIC, critical P:L ratio (P:L*), and membrane affinity(*K_p_*):

(1) $$\text{MIC}=\frac{\text{P}:{\text{L}}^{*}}{K_{p}\cdot \gamma_{\text{L}}}$$

where γ_L_ is the lipid molar volume, a known value for artificial lipid fluid bilayers (Chiu et al., 1999).

**Figure 1:**
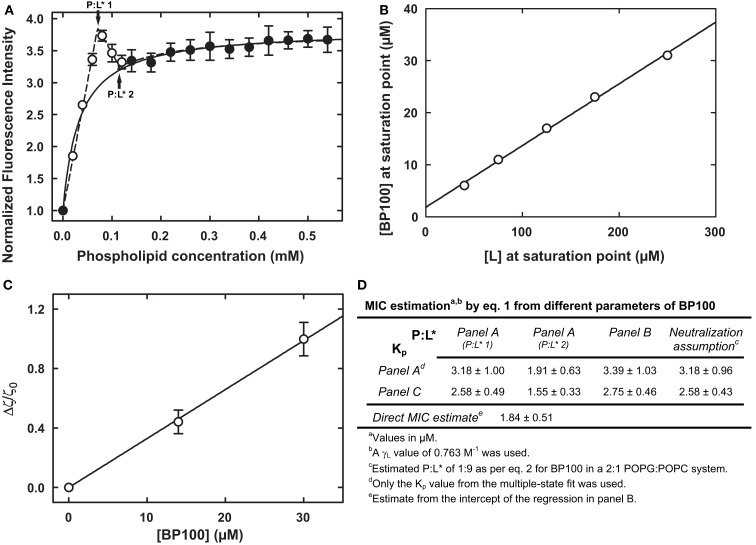
Different experimental methods to determine ***K_p_*** and P:L* for a peptide-vesicle system, exemplified with the peptide BP100 with liposomes of 2:1 anionic-to-zwitterionic lipid constitution (Ferre et al., 2009). **(A)** Partition curve with an obvious deviation from hyperbolic behavior at low lipid concentrations; a *K_p_* of (39.3 ± 14.4) × 10^3^ was obtained from a simple fit to the filled data points (Ferre et al., 2009); a fit to all data points was possible by assuming the coexistence of different bound states (Melo and Castanho, 2007), yielding a *K_p_* of (45.8 ± 13.8) × 10^3^ and two P:L* values, of 0.111 ± 0.01 and 0.067 ± 0.008, respectively (indicated by arrows). **(B)** Membrane saturation points obtained at different peptide and lipid concentrations; the linear fit (Ferre et al., 2009) yielded a P:L* of 0.118 ± 0.003; the intercept of (1.84 ± 0.51) μM is a direct estimate of the MIC as per Eq. 1 (Melo et al., 2011). **(C)** Normalized ζ-potential measurements from where a *K_p_* of (56.4 ± 9.4) × 10^3^ was extracted (Freire et al., 2011). **(D)** Summary of the possible MIC calculations, by Eq. 1, from different parameters (estimated – see Eq. 2 – or from panels **A–C**); these overlap nicely with the 2.5–7.5 μM range where BP100 is active against different Gram-negatives (Ferre et al., 2009; Alves et al., 2010).

Equation 1 does require the determination of the P:L* and *K_p_* parameters. We provide here a summary of methods to their determination using model membranes, as well as collected examples with the peptide BP100 in Figure 1:

#### Partition data

The value for *K_p_* can be extracted from experiments where the fraction of bound peptide is determined at different concentrations of lipid (Figure 1A; see Santos et al., 2003, for an overview on *K_p_* extraction from these curves). Oftentimes data points at low lipid concentrations – the conditions at which the peptide is most concentrated in the membrane – will deviate from the expected hyperbolical relationship. By fitting a model that accounts for different membrane-bound states (Melo and Castanho, 2007) a value for *K_p_* and two critical P:L ratios can be recovered (Figure 1A).

Other setups similar to the described above, but not necessarily involving a lipid titration, can be used to determine *K_p_*: isothermal titration calorimetry (Bastos et al., 2008) is an accurate alternative; peptide quantification after separation by ultracentrifugation has also been successfully applied (Cirac et al., 2011); and a method has been recently put forth that allows *K_p_* extraction from ζ-potential data (Figure 1C; Freire et al., 2011).

#### Threshold points

There is a linear relationship between global peptide and lipid concentrations for a threshold point to be reached (Figure 1B; Pott et al., 1998; Melo and Castanho, 2007). P:L* can be recovered from the slope of this peptide vs. lipid relationship, whereas the intercept will be itself a direct estimate of the MIC (this is so because the limit of zero lipid concentration parallels the very low lipid concentration in a MIC assay; Melo et al., 2011).

#### Neutralization assumption

For some peptides it may be plausible to assume that P:L* coincides with, or is triggered by, electrostatic neutralization of the membrane (Ferre et al., 2009). In the case of a one-to-one peptide-lipid charge neutralization it can be seen that P:L* will be given by:

(2) $${\text{P:L}}^{\text{*}}=\frac{f_{C}\cdot Z_{L}}{Z_{P}}$$

where *f_C_* is the fraction of charged lipids, *z_L_* the absolute formal charge per charged lipid, and *z_P_* the absolute formal charge on the peptide (assuming peptide and lipid charges have opposite signs; extra terms must be introduced in case of multiple lipids of different charges). This assumption provides an easy shortcut to a potentially relevant threshold, if no other data are available.

Equation 1 is not only simple and straightforward to use, but was also successfully applied to data for peptides BP100 (Figure 1D) and omiganan (Melo et al., 2011). Furthermore, it should be remarked that although the involved research was spurred by such observations the applicability of Eq. 1 does not depend on P:L* actually being very high. Nor is Eq. 1 restricted to bacterial death: it may be applicable to systems with relevant membrane threshold events, such as hemolysis (Melo et al., 2011).

### Improving the MICs of AMPs

It is interesting to analyze the issue of AMP activity optimization under the light of Eq. 1. It can be seen that lower MICs can be achieved either by having peptides with a low P:L* (i.e., requiring very little membrane-bound concentration to trigger bactericidal action) or with a high *K_p_* (i.e., having a very strong affinity for the membrane). Designing peptides with lowered P:L* is not straightforward as the cooperative mechanisms involved in disruption are not yet fully understood. On the other hand, improving *K_p_* is an apparently easier task: one would need only increase the charge density on the peptide as much as possible, which will then boost the affinity for the anionic bacterial membrane. However, peptide charge density is a double-edged sword: too much of it will cause surface neutralization at low bound concentrations – perhaps too low to trigger activity (Alves et al., 2010) – and subsequent peptide binding will no longer be favored after the electrostatic driving forces have been quenched. Given this, it may be the case that typical AMPs have evolved to have the highest charge density that does not compromise their accumulation in the membrane. Surface charge neutralization at membrane disruption would then arise not from a cause-effect relationship but rather as a consequence of optimal antibacterial peptide properties.

## Conclusion

The path that bridges biophysical data with macroscopic physiological observations is paved with assumptions that are sometimes taken for granted. We started this work by challenging the notion of unphysiologically high AMP P:L ratios. By then taking a modeling approach we not only demystified the issue, but also achieved new perspectives on AMP action that ultimately led to a predictive model – interestingly, one that does not require specifically high P:L ratios and is therefore even compatible with more conservative views.

Just as there are exceptions to almost any characteristic a typical AMP is said to possess, so do we expect there to be several cases of peptides that will not conform to our predictions. At any rate, a simple new approach to AMP action is provided that we hope will spur novel developments on the field.
